# Report of an Interesting Case

**Published:** 1903-04

**Authors:** O. L. Holmes

**Affiliations:** Stewart, Ga., Ex-House Surgeon, Grady Hospital


### REPORT OF AN INTERESTING CASE.

By O. L. HOLMES, M.D., STEWART, GA.,
Ex-House Surgeon, Grady Hospital.

The case was one of pregnancy complicated with albuminuria and mitral regurgitation. I was called to see patient on 18th of April, 1902. Found a primipara eighteen years old, well developed, with history of pregnancy existing since the preceding November; was very anemic, pulse irregular and temperature normal, urine scant and high colored, feet and legs swollen and edematous, and external genitals swollen. Said she had been this way for ten days or two weeks. On examination I found the external organs swollen and filled with an effusion, very tense, painful to touch, and I was unable to pass finger into vaginal canal. On examination of heart, found a well marked mitral regurgitant murmur. Placed her in the dorsal position across bed and punctured the labia in numerous places and had hot applications applied constantly. Ordered calomel grs. iii for her, to be followed with magnesium sulphate ℥ss in eight hours. Placed her on strychnin sulph. gr. 1-30 every four hours and tr. digitalis gtts. x three times a day. Carried specimen of urine to office for examination; found it loaded with albumin. No microscopical examination made.

In about four hours I was hurriedly called, the messenger telling me she was dying.

I found patient in a comatose condition, pulse imperceptible and slight convulsive movements of muscles of body. I began stimulating with strychnin sulph. gr. 1-30 every hour, and whiskey hypodermically every ten minutes, and had heat applied constantly. This condition lasted for three or four hours, when she began to rally. I then told her husband and father, who were present, that if she revived sufficiently I was going to anesthetize her and try to bring on a miscarriage. Explained to them the dangers of doing so, and of waiting; they wanted me to go ahead and do what I thought best. After about another hour she had revived sufficiently for me to make an attempt to produce a miscarriage.

Placing her in the dorsal position across the bed, I began the anesthesia with chloroform, to which she responded rapidly and without any bad effects. I turned the anesthetic over to an assistant (who was inexperienced). After cleansing my hands and the parts I attempted an examination. I found the vaginal walls filled with an effusion so much so that it was difficult to pass my finger between them. I was unable to distinguish the uterus, so I had to give up the idea of producing a miscarriage; not only from the fact that I was unable to distinguish the uterus, but also from the fact that the passage of the fetus at this time would cause a severe laceration of all the parts. Left off the anesthetic, and after she had rallied from its effects, she was apparently a good deal better. Pulse was stronger and the convulsive movements less marked.

I placed her on the following treatment and left for home, telling them to notify me of any change.

The treatment was as follows:

Strychnin sulph. gr. 1-30 every three hours.

℞ Bromide soda.......................................gr. xv
Chloral hydrate.........................gr. viiss. to the ʒ
Every hour until sleep was produced.

Saw her next morning and her condition was greatly improved, pulse good, temperature normal, laughing and talking with her husband. She slept well the latter part of the night.

I continued the strychnin every four hours. Tincture digitalis gtts. x three times a day, chloroform gtts. x every four hours, and placed her on:

℞ Calomel powder.
Squills "
Digitalis " .....................................aa gr. i
M. and fix capsule i.
Sig.: One every other night, followed in the morning with magnesium sulphate ℥ss, and repeated if necessary.
Also on acetate of potash gr. xv to the ʒ every four hours.

After the third day she began improving, and the improvement was steady.

Urine was examined every third day, showing about the same amount of albumin for the first three or four examinations, when it began decreasing, the swelling gradually diminished, patient said she had not felt the movements of the child in three to four weeks, and on examination I was unable to distinguish the fetal heart beat. On the fourth of May following, she began having slight labor pains, which continued until the 5th, when they increased in severity. I was called about 5 A.M. on the 5th, and in an hour after my arrival she was delivered of a six-months fetus, fully developed, weighing five and a half pounds, which had evidently been dead for about three or four weeks. Placenta came away without any interference. I washed the uterus out with a two per cent. carbolic solution, discontinued all the medicine except the acetate of potash, and placed her on tonic treatment, using the elixir of iron, quinin and strychnin. The albumin cleared up and she improved rapidly. Kept her in bed sixteen days, after which I allowed her to sit up and gradually to get out.

The patient regained her strength rapidly, has had no trouble since, and expects to be confined in April. She still has a slight mitral regurgitant murmur, but is the picture of health otherwise.

